# An adaptive gene-level association test for pedigree data

**DOI:** 10.1186/s12863-018-0639-2

**Published:** 2018-09-17

**Authors:** Jun Young Park, Chong Wu, Wei Pan

**Affiliations:** Division of Biostatistics, University of Minnesota, 420 Delaware Street SE, Minneapolis, MN 55455 USA

**Keywords:** aSPU, GWAS, HDL, Linear mixed models, Score test

## Abstract

**Background:**

We propose a gene-level association test that accounts for individual relatedness and population structures in pedigree data in the framework of linear mixed models (LMMs). Our method data-adaptively combines the results across a class of score-based tests, only requiring fitting a single null model (under the null hypothesis) for the whole genome, thereby being computationally efficient.

**Results:**

We applied our approach to test for association with the high-density lipoprotein (HDL) ratio of post- and pretreatments in GAW20 data. Using the LMM similar to that used by Aslibekyan et al. (PLos One, 7:48663, 2012), our method identified 2 nearly significant genes (*APOA5* and *ZNF259*) near rs964184, whereas neither the other gene-level tests nor the standard test on each individual single-nucleotide polymorphism (SNP) detected any significant gene in a genome-wide scan.

**Conclusions:**

Gene-level association testing can be a complementary approach to the SNP-level association testing and our method is adaptive and efficient compared to several other existing gene-level association tests.

## Background

Genome-wide association studies (GWASs) are considered to be the standard approach to use to detect common genetic variants associated with complex traits. It has become popular to extend the most popular single-nucleotide polymorphism (SNP)-level analysis to gene-level analysis by aggregating multiple SNPs in a gene or other functional unit. As a complement to the standard single SNP-based approach, the gene-level approach can achieve higher reproducibility and power. An additional benefit of the gene-level approach is that a decreased number of hypotheses need to be tested, thereby reducing the burden of multiple testing.

The goal of this work is to perform a gene-level association test to detect genes significantly associated with a single trait using the GAW20 data while effectively controlling for the false-positive rate. Note that the candidate gene approach conducted by Aslibekyan et al. was based on the 95 loci drawn from previous studies based on SNP-level association testing [1], and found SNP rs964184 to be strongly associated with the high-density lipoprotein (HDL) ratio of post- and pretreatments. We are interested in determining whether a gene-level analysis can lead to uncovering significantly associated genes, and, in particular, whether the genes near rs964184 are significantly associated in a genome-wide scan. Specifically, we apply the adaptive sum of powered score (aSPU) test [2], which is motivated to account for unknown and varying association patterns (eg, varying numbers or proportions of associated SNPs) across the genes, thus maintaining higher power than other nonadaptive gene-level tests. The aSPU test is computationally feasible as it does not require to fit separate models for each SNP or gene, and it satisfactorily controls false-positive rates. Note that the aSPU test was originally proposed for generalized linear models, and extended to generalized estimating equations and generalized linear mixed models (GLMM) [3–5]. Its application to and empirical performance in linear mixed models (LMMs), especially with large pedigree data, have not been discussed in previous studies.

The Genetics of Lipid Lowering Drugs and Diet Network (GOLDN) study collected pedigree data, motivating the use of LMMs to account for population structures and relatedness as adopted by Aslibekyan et al. [1]. In our LMM, we account for genetic relatedness among subjects as a random effect with a covariance matrix calculated based on individual-level SNP data. We also adjusted for covariates such as age gender, and study center. In this paper, we present the results of the aSPU test based on LMM and compare with other existing gene-level tests and individual SNP analysis.

## Methods

Suppose that *y*_*i*_ denotes a quantitative trait for individual *i* = 1, ⋯, *n*, ***X***_*i*_ ***=*** (*X*_*i*1_, ⋯, *X*_*iq*_)^′^ is a vector of *q* covariates, and ***G***_*i*_ ***=*** (*G*_*i*1_, ⋯, *G*_*ip*_)^′^ is a vector of *p* SNPs in a gene for individual *i*. A LMM is constructed as

1 $$ {y}_i={\boldsymbol{X}}_i\boldsymbol{\alpha} +{\boldsymbol{G}}_i\boldsymbol{\beta} +{b}_i+{\varepsilon}_i $$

where ***α*** and ***β*** are the unknown regression coefficient vectors for the corresponding covariates and SNPs, *b*_*i*_ and *ε*_*i*_ are a random intercept and an error term that are independent with each other. We further assume that the error terms *ε*_*i*_s are independently distributed, but *b*_*i*_s are not. Specifically,

2 $$ \boldsymbol{b}={\left({b}_1,\dots, {b}_n\right)}^{\prime}\sim \mathcal{N}\left(\mathbf{0},\tau \cdot \boldsymbol{\Psi} \right)\mathrm{and}\kern0.3em \boldsymbol{\varepsilon} ={\left({\upvarepsilon}_1\dots, {\upvarepsilon}_n\right)}^{\prime}\sim \mathcal{N}\left(\mathbf{0},\phi \cdot \boldsymbol{I}\right) $$

where **Ψ** is a known *n × n* genetic relationship matrix, which reflects the genetic relatedness among the subjects in the data. The null hypothesis to be tested for association between the group of the SNPs and the trait is *H*_0_ : ***β*** = **0**.

Fitting (generalized) LMMs can be computationally demanding. However, using penalized quasi-likelihood (PQL) to fit the model enables us to extract the test statistic for score-based tests including the aSPU test [6]. It is known that maximizing PQL is equivalent to maximizing the likelihood for quantitative traits. Specifically, we first need to fit the LMM under the null hypothesis.

3 $$ {y}_i={\boldsymbol{X}}_i\boldsymbol{\alpha} +{b}_i+{\varepsilon}_i, $$

from which, the score vector ***U*** = (*U*_1_, ⋯, *U*_*p*_)^′^, to be used to construct various gene-level score-based tests, can be expressed as

4 $$ {U}_j={\sum}_{i=1}^n{G}_{ij}\left(\frac{y_i-\left({\boldsymbol{X}}_i\hat{\boldsymbol{\alpha}}-{\hat{b}}_i\right)}{\hat{\phi}}\right) $$

The aSPU test statistic can be obtained using the score vector ***U*** and its covariance matrix ***V*** under the null hypothesis, which can also be written in a closed form. Because the score vector follows asymptotic normal distribution with mean zero under the null hypothesis, one can use the Monte Carlo method to compute *p*-values. Note that both ***U*** and ***V*** depend only on the null model (3), which provides computational efficiency when the number of tests is large as in a genome-wide scan. We can use an R package *GMMAT* to derive ***U*** and ***V*** [7].

We briefly introduce the idea of the aSPU test here. All score-based association tests require ***U*** and ***V***, and each nonadaptive test has its own advantages and disadvantages. For example, consider these 2 cases: (a) every SNP encoded in a gene is associated with an equal effect size and direction, and (b) only one or a small proportion of the SNPs are associated. The burden test, which takes ${\sum}_{j=1}^p{U}_j$ as a test statistic, is desired in the first case, but it will lose power in the second case. On the other hand, the UminP test, which takes max{|*U*_1_|, ⋯, |*U*_*p*_|} as a test statistic when the variances of the score elements are the same, is advantageous in the second case but not in the first case. Thus, applying a single and nonadaptive score-based test might not be powerful in gene-level analysis. The aSPU test offers a way to combine various score-based tests; it is based on a class of the sum of powered score (SPU) tests indexed by a positive integer *γ*. Specifically, the SPU(*γ*) test statistic is.

5 $$ {T}_{SPU\left(\gamma \right)}={\sum}_{j=1}^p{U}_j^{\gamma}\mathrm{and}\;{T}_{SPU\left(\infty \right)}=\max \left\{\left|{U}_1\right|,\dots, \left|{U}_p\right|\right\} $$

It is easy to see that the burden test and the sum of squared score (SSU) test are equivalent to the SPU(1) and SPU(2) tests respectively. It was also shown that SPU(2) is equivalent to sequence kernel association test (SKAT) with the linear kernel and to Multivariate Distance Matrix Regression (MDMR) with the Euclidean distance (under the framework of LMM) [8]. Furthermore, assuming the equal variance of the score elements, the UminP test is equal to SPU test with *γ* = ∞. One can treat *γ* as a factor that decides the weight on each score element. The aSPU test uses the minimum *p* value of the SPU tests as the test statistic, which provides a general data-adaptive method to test for associations. The set of *γ* ∈ {1, 2, ⋯, 8, ∞} was proposed by Pan et al. based on experiences [2].

## Results

The LMM we used for the GAW20 data was similar to that used by Aslibekyan et al.; we used the ratio of post- and pretreatment HDL as the trait, and we used age, gender, and study center as covariates. The only difference was the covariance matrix of the random effects. Our covariance matrix **Ψ** of the random effects reflected the genetic relatedness, where each **Ψ**_*ij*_ was the Pearson correlation coefficient between 2 subjects *i* and *j* of 20,000 randomly selected SNPs. Our analysis was based on 821 subjects who did not have missing values in either the trait or the covariates. We only included common variants with minor allele frequencies (MAFs) greater than 0.05. Among those, we randomly imputed missing variants using MAF if the proportions of missing values were less than 1%. It resulted in a total of 595,304 SNPs included in our analysis. For the gene-level analysis, we used hg18 as a reference genome and each gene included the SNPs that were within 10,000 regions upstream or downstream of the gene’s coding region. In total, we included 22,434 genes in our analysis.

**Fig. 1:**
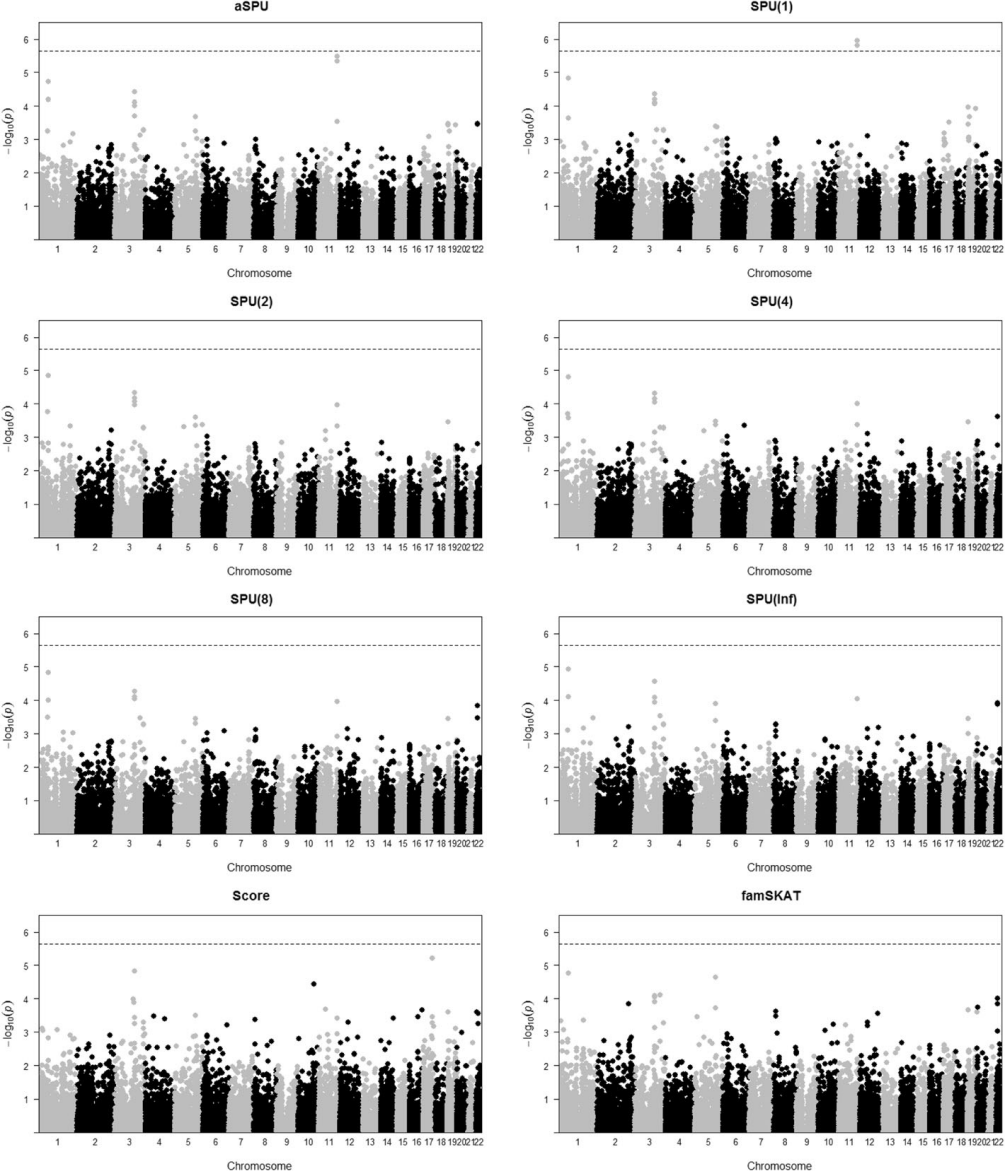
The Manhattan plots for the gene-level association tests

**Fig. 2:**
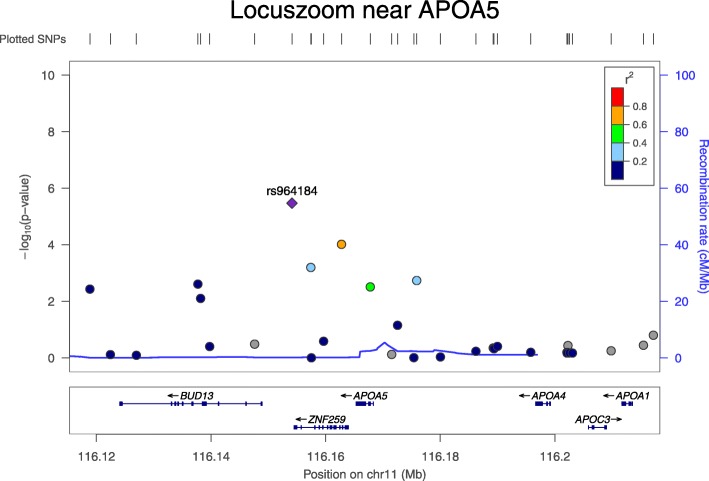
The LocusZoom plot near *APOA5* gene

We conducted the SPU(*γ*) and aSPU tests under the LMM. In addition to the SPU(1), SPU(2), and SPU(∞) tests where their theoretical equivalences with other existing gene-level tests are shown in the Methods section, we also performed the gene-level score test and the famSKAT (family-based sequence kernel association test) [9] using the same covariates and relationship matrix. Figure 1 shows the results of the tests. Using the Bonferroni adjustment for the genome-wide significance level (α = 0.05), the aSPU test and the score test did not detect any significant genes, but 2 genes (*APOA4* and *ZNF259* on chromosome 11) were close to being significant. However, these 2 genes were detected by the SPU(1) test, suggesting that their association effects were not dominated by a small number of variants. We emphasize the adaptiveness property of the aSPU test by noting gene *BUD13* on chromosome 11 and *GUCD1* and *SNRPD3* on chromosome 22, whose −log_10_(*p* values) were not less than 3 by SPU(1), but much larger by the SPU(∞) test (as well as by a few other SPU tests and the aSPU test). We also note that *APOA5* and *ZNF259* were located nearby as shown in Fig. 2. In particular, they shared 7 variants out of 9 SNPs in both genes. The gene-level score test yielded a gene (*DDX42* on chromosome 17) almost significant at the genome-wide significance level, but the score test did not detect any loci near rs964184. Similarly, the famSKAT did not detect any significant gene.

<insert Figure(s) 1 and 2 here>.

**Fig. 3:**
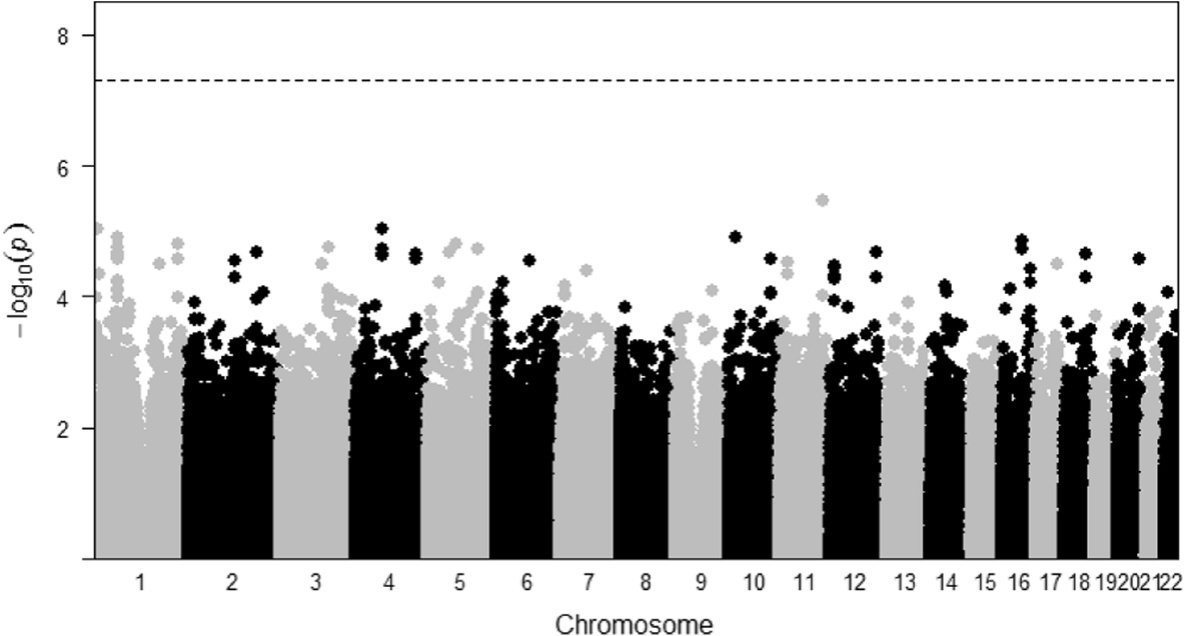
The Manhattan plot for the SNP-level association test

We also compared the gene-based tests to the score test for single variants. We used the usual 5 × 10^− 8^ as the genome-wide significance level for the SNP-level analysis. Even though rs964184 turned out to be the one most significantly associated with the trait among all the SNPs, its *p* value was far away from the genome-wide significance level, as shown in Fig. 3. This example partially confirms the usefulness of gene-level testing.

## Discussion

In GWAS, individuals in pedigree data are not independent, thus motivating the use of (generalized) LMMs. We considered a general LMM with a random intercept that reflects the genetic relatedness among the subjects. We then conducted the aSPU test on the genes across the whole genome based on fitting a single null model, and identified 2 genes near SNP rs964184 to be nearly significant. In contrast, none of the SNPs, including SNP rs964184, were nearly significant in a standard single SNP-based analysis.

## Conclusions

We have demonstrated the applicability and usefulness of our proposed aSPU test in LMMs for association analysis of large pedigree data. Furthermore, our study has confirmed possible advantages and complementary roles of gene-level analyses with the adaptive aSPU test when compared to standard single SNP-based analyses.

## Abbreviations

aSPU: Adaptive sum of powered score
GLMM: Generalized linear mixed model
GWAS: Genome-wide association study
LMM: Linear mixed model
MAF: Minor allele frequency
SNP: Single nucleotide polymorphisms
SPU: Sum of powered score.
